# Assessment of Residual‐Serum SARS‐CoV‐2‐N‐Antigen Testing for Hospital Surveillance in Germany

**DOI:** 10.1002/jmv.71055

**Published:** 2026-07-07

**Authors:** Jasmin Weninger, Aleko Zedginidze, Antonios Katsounas, Michael Pohl, Abdurrahman Coskun, Roman Wölfel, Mustafa Özcürümez

**Affiliations:** ^1^ Department of Medicine Knappschaft Kliniken University Hospital Bochum, Ruhr University Bochum Bochum Germany; ^2^ Department of Medical Biochemistry, School of Medicine Acibadem Mehmet Ali Aydinlar University Istanbul Turkey; ^3^ Bundeswehr Institute of Microbiology Munich Germany

**Keywords:** COVID‐19, ELISA, hospital surveillance, nucleocapsid antigen, residual serum, SARS‐CoV‐2

## Abstract

Routine SARS‐CoV‐2 surveillance in hospitals is crucial for preventing in‐hospital transmission, especially among elderly and multimorbid patients. While RT‐PCR testing is sensitive, it is resource‐intensive, and rapid antigen tests lack sensitivity. This study evaluates the diagnostic performance and utility of serum N‐antigen ELISA for detecting active SARS‐CoV‐2 infection in hospitalized patients during the Omicron period. A prospective, non‐interventional diagnostic accuracy study was conducted at a tertiary‐care university hospital in Germany from 6 January to 8 February 2023. Residual serum samples were tested for N‐antigen using a commercial ELISA and compared with RT‐PCR results from respiratory swabs. Diagnostic latency, sensitivity, specificity, and predictive values were calculated at the sample and patient levels. Among 2030 inpatients (12,558 patient‐days), 610 were screened by ELISA. Sensitivity and specificity were 60.6% and 99.9%, respectively, increasing to 89.6% and 99.9% for samples with ≥ 10^4^ genome equivalents/mL. Early infection showed higher sensitivity (78.6%) compared to late infection (25.5%). In a paired same‐cohort PCR‐only scenario, the combined PCR plus available serum N‐antigen workflow reduced unknown‐risk time by 243 patient‐days, corresponding to 1.94 fewer unknown‐risk days per 100 cohort patient‐days (95% bootstrap CI, 1.38–2.57). Residual‐serum SARS‐CoV‐2 N‐antigen testing offers high specificity and early‐phase sensitivity, complementing PCR‐based hospital surveillance without additional sampling. This approach may enhance the efficient detection of unsuspected infections in routine inpatient care.

**Abbreviations**
Ag: antigen
CI: confidence interval
COVID‐19: coronavirus disease 2019
Ct: cycle threshold
ECI: Elixhauser Comorbidity Index
ELISA: enzyme‐linked immunosorbent assay
GE: genome equivalents
ICU: intensive care unit
ID: identification
IQR: interquartile range
LoQ: limit of quantification
LR+/LR−: positive/negative likelihood ratio
N: nucleocapsid
NPV: negative predictive value
RAT: rapid antigen test
RT‐PCR: real‐time reverse transcription polymerase chain reaction
ROC: receiver operating characteristic
PPV: positive predictive value
S: surface
SARS‐CoV‐2: severe acute respiratory syndrome coronavirus 2
SD: standard deviation
STARD: standards for reporting of diagnostic accuracy studies

## 1 Introduction

Coronavirus disease 2019 (COVID‐19), caused by severe acute respiratory syndrome coronavirus 2 (SARS‐CoV‐2), remains a significant public health challenge, particularly in hospitals where vulnerable patients (elderly, multimorbid, or immunocompromised), face a higher risk of severe disease and outcomes. Timely detection of SARS‐CoV‐2 in hospitalized patients is essential for initiating appropriate therapy, preventing nosocomial transmission/outbreaks, and safeguarding healthcare workers and infrastructure.

Current diagnostic strategies primarily rely onreal‐time reverse transcription polymerase chain reaction (RT‐PCR) testing of nasopharyngeal or oropharyngeal swabs, typically performed at admission and during hospitalization, when indicated. Rapid antigen tests (RATs) may support faster decision‐making [1, 2, 3].

Initially, testing targeted symptomatic individuals, but this approach is insufficient, as Omicron variants and vaccinated or previously infected individuals may be asymptomatic or show nonspecific symptoms, presenting a risk for unrecognized transmission [4]. Thus, effective surveillance must detect asymptomatic or oligosymptomatic cases, especially in vulnerable populations. However, surveillance capacity is often limited by testing resources, infrastructure, and human factors in clinical practice, requiring diagnostic accuracy, procedural efficiency, and scalability.

Although RT‐PCR is highly sensitive, its interpretation is complicated by prolonged viral RNA detection beyond the infectious period, and repeated testing remains resource‐intensive in routine clinical practice [5, 6]. RATs provide faster and more cost‐effective results but lack the sensitivity to detect asymptomatic or early infections [7, 8]. This highlights the need for additional diagnostic tools that balance accuracy, timeliness, and feasibility for hospital settings.

Serum antigen testing, which detects SARS‐CoV‐2 N‐protein (antigenemia) in blood, offers an alternative. It has been associated with viral load and disease severity and may appear early in infection, even in asymptomatic cases [9, 10, 11, 12]. Serum antigen assays have shown high diagnostic accuracy for acute infection, with some studies reporting 91.7% sensitivity and 98.0% specificity for N‐antigen Enzyme‐linked Immunosorbent Assay (ELISA) compared to nasal RT‐PCR [5]. However, antigenemia is usually transient, reflecting active viral replication, and resolves within 2 weeks, distinguishing acute from residual infection, but complicates detection in convalescent patients [11, 13, 14, 15].

Serum N‐antigen testing offers a complementary tool for hospital surveillance, using residual serum from routine blood draws without burdening patients or staff. This can detect infections in patients not routinely tested by PCR, especially in settings where universal screening is no longer standard practice.

In this study, we evaluate the utility of a commercial ELISA for serum SARS‐CoV‐2 N‐antigen detection in inpatient surveillance. We conducted a prospective, non‐interventional diagnostic accuracy study at a university hospital to evaluate serum SARS‐CoV‐2 N‐antigen ELISA as an add‐on to routine inpatient surveillance. Residual serum samples obtained during routine care were prospectively archived and tested after completion of sample collection in blinded batches, with results compared against RT‐PCR from respiratory swabs.

The primary objective was to assess the diagnostic performance of serum N‐antigen detection for identifying active SARS‐CoV‐2 infection under routine inpatient‐care conditions. The secondary objective was to evaluate the feasibility of integrating RT‐PCR with serum antigen testing, without additional sampling or workflow changes beyond standard care. We also explored the temporal relationship and diagnostic concordance between antigenemia and RT‐PCR results, aiming to inform efficient surveillance strategies in the post‐peak pandemic phase.

## 2 Methods

### 2.1 Study Design and Setting

This single‐centre, prospective, non‐interventional diagnostic accuracy study was conducted at a tertiary‐care university hospital in Germany (500 beds) over 34 days (6 Jan–8 Feb 2023). The prospective components comprised the predefined observation period, eligible inpatient cohort, routine clinical follow‐up during hospitalization, daily identification and archiving of available residual serum samples from routine blood draws, and comparison with the existing RT‐PCR surveillance strategy. The serum SARS‐CoV‐2 N‐antigen ELISA index test was performed without additional phlebotomy after completion of routine diagnostics as a blinded, batched analysis of stored residual serum specimens. ELISA results were generated for research purposes only and did not influence diagnostic or therapeutic management.

### 2.2 Patients

All adult inpatients with at least one residual serum sample during the observation period were eligible, irrespective of symptoms or ward. Admissions or discharges outside the period were permitted. The final cohort comprised 2030 patients contributing 12,558 patient‐days.

### 2.3 Specimen Collection and Laboratory Methods

#### 2.3.1 RT‐PCR on Respiratory Swabs (Reference Test)

Combined nasal/oropharyngeal swabs were obtained at admission and approximately every 4 days or as clinically indicated. Swabs in Cobas® PCR Media Uni Swab were analysed on the cobas® 6800 platform (Roche Diagnostics, Basel, Switzerland), with a cycle threshold (Ct) ≤ 40 considered positive. Viral RNA load was expressed as genome equivalents per mL (GE/mL) using site‐specific standard‐curve conversion from Ct values. Patients with ≥ 1 positive RT‐PCR were classified as infected for patient‐level analyses. Variant‐screening RT‐PCRs (TibMolBiol, Berlin, Germany) were used according to contemporaneous surveillance needs, but not for outcome definition.

#### 2.3.2 Serum N‐Antigen ELISA (Index Test)

Residual serum was aliquoted after routine testing and stored at −20°C for batch analysis using the commercial CE‐marked ScheBo® SARS‐CoV‐2 Antigen ELISA, Article No. 33, ScheBo Biotech AG, Giessen, Germany, according to the manufacturer's instructions. ELISA testing was started approximately 30 days after the end of the observation period and completed within 1 week; frozen storage therefore lasted approximately 30–70 days, depending on the individual sample collection date and testing day. Aliquots were thawed once, mixed, centrifuged, and analysed manually in duplicate by trained laboratory staff. Absorbance was read on a Tecan Infinite F50 microplate reader at 450 nm with a 620 nm reference wavelength, and concentrations were calculated from the calibrator curve. The limit of quantification (LoQ) was 2.89 pg/mL, the predefined positivity cut‐off was 2.97 pg/mL, and the upper quantification limit was 180.01 pg/mL (samples above were diluted 1:10 and re‐assayed). Grossly haemolytic/lipemic/clotted samples were excluded. Because analytical sensitivity declines at low viral burdens, performance summaries are presented overall and restricted to samples with RT‐PCR viral load ≥ 10^4^ GE/mL.

### 2.4 Outcomes and Definitions

#### 2.4.1 “Unknown‐Risk” Days

“Unknown‐risk” days were defined as inpatient calendar days during the observation period on which no SARS‐CoV‐2 result from RT‐PCR or serum N‐antigen ELISA was yet available for the respective patient. Counting started on the first observed inpatient day within the study period and ended on the calendar day of the first available SARS‐CoV‐2 result. Because exact result times within a calendar day were not evaluated, the day of first testing was counted as an unknown‐risk day. For patients without any available SARS‐CoV‐2 result, all observed inpatient days during the study period were classified as unknown‐risk days.

For each patient, unknown‐risk days were calculated separately for the observed combined workflow and for the same‐cohort PCR‐only scenario, in which serum N‐antigen results were ignored while the observed PCR dates and inpatient follow‐up were retained.

#### 2.4.2 Testing Modality

Patients were categorized as PCR‐only, ELISA‐only, both, or neither. These modality‐defined categories were treated as descriptive workflow strata and were not interpreted as randomized comparison groups.

#### 2.4.3 First‐Result Attribution

For patients with both modalities, the earliest result was defined as PCR‐first, ELISA‐first, or same‐day (if results occurred on the same date; time of day was not considered).

#### 2.4.4 Diagnostic Accuracy

For sample‐based analysis, each ELISA measurement was paired to the closest RT‐PCR within 24 h (ties resolved to the earlier RT‐PCR). In case of patient‐based analysis, reference‐positive was defined as any positive RT‐PCR during hospitalization; index‐positive as any positive ELISA during hospitalization. Diagnostic sensitivity, specificity, positive predictive value (PPV), negative predictive value (NPV), correctly classified proportion, positive and negative likelihood ratios (LR+/LR−) were computed overall and for samples with RT‐PCR viral load ≥ 10^4^ GE/mL.

#### 2.4.5 Phase of Infection for False Negative Analyses

For patients with ≥ 2 RT‐PCRs, the viral load trajectory (log_10_ GE/mL over time) was classified as early (increasing), peak, or late (decreasing). Each ELISA result was assigned to a phase based on its timing relative to flanking RT‐PCRs (categorized as early/peak if it fell during an increasing or peak viral load period, or late if during a declining phase).

#### 2.4.6 Handling of Discordances and Artefacts

ELISA‐positive/RT‐PCR–negative discordances were individually reviewed for pre‐analytical error. One suspected mislabel was treated as an artefact and excluded from specificity estimates.

### 2.5 Statistical Analysis

Coverage was summarized at patient and patient‐day levels. Unknown‐risk days were reported as absolute counts, within‐group rates per 100 patient‐days, and cohort‐level shares. Modality‐defined groups were treated as descriptive workflow strata and were not interpreted as randomized comparison groups. To estimate the incremental contribution of serum N‐antigen testing, we performed a paired same‐cohort PCR‐only scenario analysis. For each patient, the observed combined PCR plus available serum N‐antigen workflow was compared with a scenario in which serum N‐antigen results were ignored while the same admission dates, inpatient follow‐up, and PCR result dates were retained. Patient‐level bootstrap resampling with 10,000 replicates was used to derive 95% confidence intervals for unknown‐risk days and rate differences. Time to first available SARS‐CoV‐2 result was modelled using a paired scenario Cox model with robust standard errors clustered by patient. The adjusted model included clinical discipline and intensive care unit (ICU) status at first ward assignment. Hazard ratios greater than 1 indicate faster result availability.

Correlation between quantitative serum N‐antigen concentration and viral load was assessed using Spearman's rank correlation among antigen‐positive RT‐PCR‐positive paired samples with numeric serum N‐antigen concentrations. Serum N‐antigen positivity was additionally modelled by logistic regression using log10 viral load as a predictor.

Receiver operating characteristic (ROC) analyses were performed using serum N‐antigen concentration as a predictor for RT‐PCR positivity and viral‐load categories ≥ 10^4^ and ≥ 10^5^ GE/mL. The manufacturer cut‐off of 2.97 pg/mL was retained as the primary operating point; antigen‐negative results without numeric concentration were assigned below this cut‐off for ROC ordering. AUCs and operating characteristics were estimated with patient‐level bootstrap 95% confidence intervals.

For accuracy analyses, sensitivity, specificity, PPV, NPV with 95% confidence intervals (CIs) (exact/Clopper–Pearson where appropriate) and LRs were calculated. Group comparisons used Fisher's exact test or *χ* ^2^ as applicable; continuous data are presented as median (interquartile range, IQR) or mean (standard deviation, SD). Two‐sided statistical tests were used, with *p* < 0.05 indicating significance. All analyses were performed in R 4.0.3.

### 2.6 Ethical Considerations

The study was approved by a local ethics committee (22‐7527) and conducted in accordance with the Declaration of Helsinki. ELISA testing was permitted only as an add‐on to RT‐PCR. The manufacturer supplied ELISA kits but had no role in study design, analysis, or interpretation. Samples were de‐identified; data processing complied with the EU General Data Protection Regulation. Methods follow Standards for Reporting of Diagnostic Accuracy Studies (STARD) recommendations [16].

## 3 Results

### 3.1 Patient Characteristics

During the 34‐day observation, 2,030 adult patients contributed 12,558 patient‐days (Table 1). The median length of stay was 5 days (IQR 3–10); mean bed occupancy was 79.4%. In‐hospital mortality was 15.3% (9/59) among PCR‐positive versus 3.9% (76/1,971) among PCR‐negative patients (risk ratio 3.96, 95% CI 2.08–7.51; odds ratio 4.49, 95% CI 2.13–9.46). Residual serum was available from 1,884 patients; 610 randomly selected patients were screened by N‐antigen ELISA. The overall median Elixhauser Comorbidity Index (ECI) was 10 (IQR 6–18), while the 85 patients deceased during observation yielded a median of 24 (IQR 16–27; *p* < 0.012).

**Table 1:** SARS‐CoV‐2 inpatient screening and sample collection, Bochum, Germany, January–February 2023 (*n* = 2,030).

Parameter	Value	%
Observation period, days	34	—
Hospitalized patients	2030	—
Patient days within the observation period	12,558	—
Occupancy rate	—	79.4
Length of hospital stay (median of days; [IQR])	5 [3–10]	—
Patients by estimated 1‐year mortality risk^a^		
High (> 15%)	1628	80.2
Moderate (5%–15%)	325	16.0
Low (< 5%)	77	3.79
Patients deceased in the hospital	85	4.19
Patients with SARS‐CoV‐2‐PCR from a swab	1756	—
RT‐PCR‐tested patient days, *n*	3953	—
SARS‐CoV‐2‐PCR per patient (median; [IQR])	2 [1–3]	—
Patients with blood draws, including serum	1884	—
Patients screened by serum‐N‐protein ELISA	610	—
ELISA‐tested patient‐days, *n*	2158	—
Blood draws per patient (median; [IQR])	2 [1–4]	—

Abbreviation: IQR: interquartile range

^a^ Risk assessment based on each patient's age‐adjusted van Walraven Elixhauser comorbidity index [17, 18].

### 3.2 Screening Coverage and Diagnostic Latency

Among 2030 inpatients, 1190 (58.6%) underwent PCR‐only, 566 (27.9%) PCR + ELISA, 44 (2.2%) ELISA‐only, and 230 (11.3%) received neither test. An overview of the screening distribution can be found in Supplementary Figure S1. Across 12,558 cohort patient‐days, 3189 were classified as unknown‐risk days in the observed combined workflow, corresponding to 25.4 unknown‐risk days per 100 patient‐days and a mean of 1.57 unknown‐risk days per patient.

Compared with PCR‐only, with 1699 unknown‐risk days over 5982 patient‐days, i.e., 28.4 per 100 patient‐days, patients with both RT‐PCR and serum N‐antigen testing contributed 839 unknown‐risk days over 5828 patient‐days, i.e., 14.4 per 100 patient‐days. Because these modality‐defined strata were defined by observed testing workflows rather than random allocation, they were interpreted descriptively only. For ELISA‐only, 108/205 patient‐days remained unknown (52.7 per 100), while untested patients contributed 543/543 unknown‐risk days (100.0 per 100). Full metrics are shown in Table 2.

**Table 2:** SARS‐CoV‐2 inpatient testing coverage and diagnostic latency, Bochum, Germany, January–February 2023 (*n* = 2030).

Modality	Patients, *n*	Length of stay, days	Residual unknown‐risk, days	Residual unknown‐risk/100 patient‐days (within group)	Share of cohort unknown‐risk days, %
Neither	230	543	543	100	4.32
ELISA‐only	44	205	108	52.7	0.86
PCR‐only	1190	5982	1699	28.4	13.5
Both	566	5828	839	14.4	6.68
Overall	2030	12,558	3189	25.4	25.4

*Note:* Unknown‐risk days were counted from admission through the day of the first available result (RT‐PCR or serum N‐antigen ELISA), inclusive of the day of testing. “Unknown‐risk/100 patient‐days” expresses residual risk within each group; “Share of cohort unknown‐risk” uses the cohort total (12,558 patient‐days) as the denominator. These strata were descriptive and were not interpreted as randomized comparison groups.

In a paired same‐cohort PCR‐only scenario, in which serum N‐antigen results were ignored while the same patients, follow‐up time, and RT‐PCR result dates were retained, the cohort would have accumulated 3432 unknown‐risk days (27.3 per 100 patient‐days) versus 3189 (25.4 per 100 patient‐days) observed, indicating that serum N‐antigen testing reduced absolute unknown‐risk time by 243 patient‐days (95% bootstrap CI 173–324) corresponding to1.94 per 100 patient‐days (95% bootstrap CI 1.38–2.57). Among the 566 dual‐tested patients, the earliest result was PCR‐first in 304 patients (473 diagnostic latency days resolved), ELISA‐first in 53 (126 days), and same‐day in 209 (240 days), showing ELISA occasionally provided the earliest actionable result. In the paired scenario Cox sensitivity analysis, the observed combined workflow was associated with faster result availability than the PCR‐only scenario (HR 1.083, 95% CI 1.064–1.101). This association remained stable after adjustment for clinical discipline and ICU status (HR 1.076, 95% CI 1.059–1.094).

### 3.3 Diagnostic Performance of ELISA

In the sample‐based analysis (Table 3) of 801 paired samples with known infection status, the apparent ELISA positivity was 7.61%, versus a true prevalence by PCR of 12.36%. The correctly classified proportion was 95.01%; sensitivity 60.61% (95% CI 50.28–70.28) and specificity 99.86% (95% CI 99.21–100). PPV 98.36% and NPV 94.73%; LR+ 425.5, LR− 0.395.

**Table 3:** Diagnostic performance of serum SARS‐CoV‐2 N‐antigen ELISA, Bochum, Germany, January–February 2023 (*n* = 801 samples; *n* = 566 patients).

Diagnostic performance characteristics	Sample‐based		Patient‐based	
	Estimate	95% CI	Estimate	95% CI
Apparent prevalence, %	7.615	5.875–9.675	3.18	1.895–4.98
True prevalence, %	12.36	10.16–14.84	10.25	7.874–13.05
Correctly classified proportion, %	95.01	93.26–96.41	92.58	90.1–94.6
Sensitivity, %	60.61	50.28–70.28	29.31	18.09–42.73
Specificity, %	99.86	99.21–100	99.8	98.91–100
Positive predictive value, %	98.36	91.2–99.96	94.44	72.71–99.86
Negative predictive value, %	94.73	92.87–96.23	92.52	89.99–94.58
Positive likelihood ratio	425.45	59.63–3035.6	148.9	20.18–1098.5
Negative likelihood ratio	0.395	0.309–0.504	0.708	0.6–0.836

Abbreviation: CI: confidence interval

In the patient‐based analysis (*n* = 566, both tests), sensitivity decreased to 29.31% (95% CI 18.09–42.73) with specificity of 99.8% (95% CI 98.91–100), PPV 94.44%, NPV 92.52%, reflecting that many infected patients never showed antigenemia at sampled time points. These results indicate ELISA's excellent rule‐in utility at high viral loads (very few false positives, high PPV), but limited rule‐out capability.

The observation period coincided with a wave predominantly driven by Omicron subvariants (Supplementary Figure S2) [19]. No major nosocomial outbreak clusters were detected; rather, infections were sporadically distributed across wards, consistent with isolated community introductions. Viral loads among PCR‐positive patients varied substantially, reflecting the dynamics of infection in a routine inpatient‐care conditions context.

### 3.4 Detection of SARS‐CoV‐2 Positive Cases

Across 99 PCR‐positive swabs with parallel ELISA testing, 60 (60.6%) were ELISA‐positive, while 39 (39.4%) were ELISA‐negative despite PCR positivity (Table 4). Most discordant samples (32/39) had ≤ 10^4^ GE/mL, referring to the ELISA's LoQ. One ELISA‐positive result without concurrent PCR was traced to a suspected pre‐analytical labelling error and treated as an artefact.

**Table 4:** Concordance of serum SARS‐CoV‐2‐N‐antigen ELISA and RT‐PCR results, Bochum, Germany, January–February 2023, at the patient‐days and patient level.

Analysis level	Serum‐N‐antigen	SARS‐CoV‐2 RT‐PCR			
		Negative	Positive	Not tested^a^	Total
Patients	Negative	507	41	44	592
	Positive	1^b^	17	0	18
	Not tested^a^	1164	26	230	1420
	Total	1672	84	274	2030
Patient‐days	Negative	701	39 (32/7)^c^	1357	2097
	Positive	1^b^	60	0	61
	Not tested^a^	2996	156	7248	10,400
	Total	3698	255	8605	12,558

^a^ not tested or no serum available, respectively.

^b^ Suspected “wrong patient in tube”.

^c^ ≤ 10^4^/ > 10^4^ GE/mL.

At the patient level, 84 patients in the full cohort had at least one positive RT‐PCR during hospitalization. Of these, 58 patients also had at least one ELISA result and were included in the patient‐level diagnostic performance analysis. Among these 58 PCR‐positive ELISA‐tested patients, 17 (29.3%) had at least one ELISA‐positive result, whereas 41 remained ELISA‐negative. The remaining 26 PCR‐positive patients had no ELISA result at the corresponding time. No ELISA antigenemia was observed among PCR‐negative patients (excluding the suspected mislabel), supporting high specificity under proper assignment but limited sensitivity for detecting all PCR‐confirmed infections.

### 3.5 Serum N‐Antigen Detectability and Viral Load

**Figure 1:**
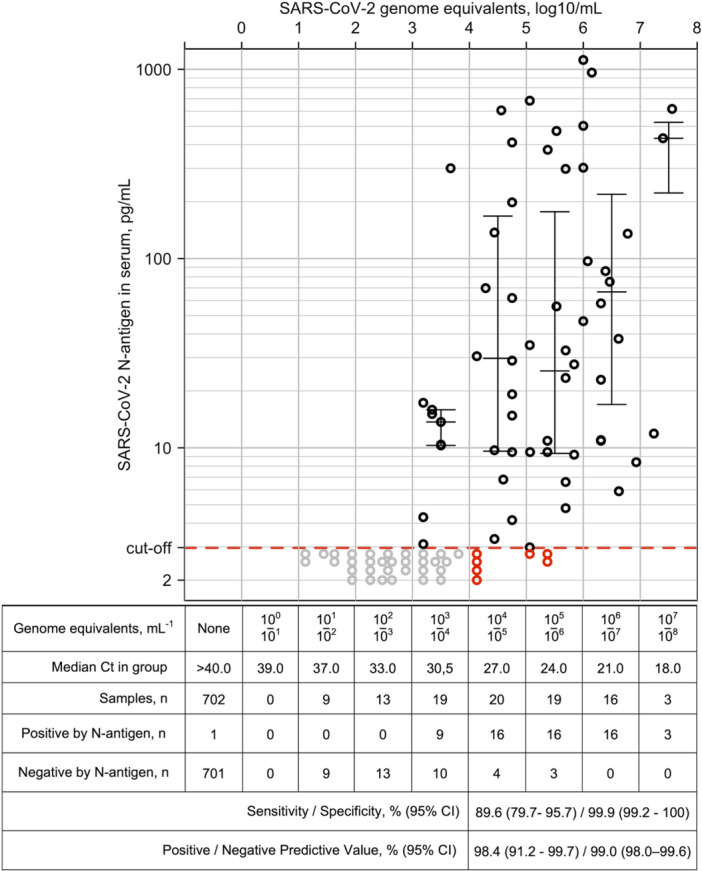
Relationship between SARS‐CoV‐2 genome equivalents and serum N‐antigen detectability, Bochum, Germany, January–February 2023 (*n* = 801 samples). Serum N‐antigen concentrations (pg/mL) are plotted against corresponding SARS‐CoV‐2 genome equivalents (log_10_/mL). Each open circle represents one sample. The red dashed line marks the assay's positivity cut‐off. Grey circles indicate samples below the positivity cut‐off, and red circles mark antigen‐negative samples despite > 4 log_10_ GE/mL detectable by PCR. For each decade, the median and interquartile range are indicated. Summary data and diagnostic performance metrics are shown in the accompanying table.

Serum N‐antigen detectability increased with SARS‐CoV‐2 viral load; median Ct declined from > 40.0 in PCR‐negative samples to 18.0 at 10^7^–10^8^ GE/mL (Figure 1). In line with assay specifications, detection was rare at ≤ 10^4^ GE/mL; all samples ≥ 10^6^ GE/mL were ELISA‐positive, while none with < 10^3^ GE/mL were positive. Negatives clustered near the assay positivity cut‐off (2.97 pg/mL). Among all RT‐PCR‐positive paired samples with serum antigen status, each 1 log10 GE/mL increase in viral load was associated with higher odds of serum N‐antigen positivity (OR 5.77, 95% bootstrap CI 3.60–18.43; *p* < 0.001; *n* = 99 samples from 38 patients). In contrast, among antigen‐positive RT‐PCR‐positive paired samples with numeric serum N‐antigen concentrations, the quantitative concentration–viral‐load association was weak (Spearman's rho = 0.23, 95% bootstrap CI − 0.14 to 0.53; *p* = 0.076; *n* = 60).

Restricting evaluation to samples ≥ 10^4^ GE/mL, sensitivity was 89.6% (95% CI 79.7–95.7), specificity 99.9% (95% CI 99.2–100), PPV 98.4% (95% CI 91.2–99.7), and NPV 99.0% (95% CI 98.0–99.6), supporting strong performance in the high‐replication range. ROC analyses showed increasing discrimination for clinically higher viral‐load endpoints, with AUCs of 0.803 for RT‐PCR positivity, 0.889 for RT‐PCR viral load ≥ 10^4^ GE/mL, and 0.936 for RT‐PCR viral load ≥ 10^5^ GE/mL. Operating characteristics at the manufacturer's cut‐off of 2.97 pg/mL are shown in Supplementary Table S1.

### 3.6 Phase‐Dependent Detection of Antigenemia

To evaluate whether serum N‐antigen detectability varied across the phase of infection, we stratified ELISA results of PCR‐positive patients into early/peak versus late phases, according to viral load trajectory (Table 5). Among early‐phase samples, 33/69 were ELISA‐positive (sensitivity 78.6%; 95% CI 63.2–89.7). In contrast, in 173 late‐phase samples, only 27 were ELISA‐positive, with dropping sensitivity to 25.5% (95% CI 17.5–39.9). Across both phases, specificity remained 100% (95% CI 96.4–100), and no false positives were observed. Phase‐stratified comparison confirmed markedly higher positivity rates in the early/peak phase (odds ratio 10.73, 95% CI 4.55–25.27; Fisher's exact *p* < 0.00001). The ECI did not differ between patients with true‐positive and false‐negative antigen results, suggesting comorbidity burden was not a determinant of detectability. Thus, antigenemia is largely confined to the initial phase of infection, providing strong rule‐in utility at high viral replication, while its diagnostic yield diminishes substantially during late viral clearance.

**Table 5:** Phase‐dependent detection of serum SARS‐CoV‐2‐N‐antigen, Bochum, Germany, January–February 2023 (*n* = 250 samples).

ELISA	Early phase (viral‐load incline)	Late phase (viral‐load decline)	Total
True positive, *n*	33	27	60
True negative, *n*	35	67	101
False negative, *n*	9	79	88
False positive, *n*	0	0	0
Sensitivity, % (95% CI)	78.6 (63.2–89.7)	25.5 (17.5–39.9)	40.5 (32.6–48.9)
Specificity, % (95% CI)	100.0 (90.0–100)	100.0 (94.6–100)	100.0 (96.4–100)

*Note:* ELISA results from 250 out of 255 patients with positive RT‐PCR either were assigned either to the early phase/peak of the infection or to the late phase, i.e., decline of viral load. (Phase comparison, i.e., ELISA positivity among PCR‐positive measurements, early *vs.* late odds ratio = 10.73, 95% CI 4.55–25.27; Fisher's exact *p* < 0.00001 (two‐sided).

## 4 Discussion

This study demonstrates that serum SARS‐CoV‐2‐N‐antigen ELISA can be implemented at scale with high specificity, good operational feasibility, and measurable system‐level benefits. While sensitivity was lower than RT‐PCR, the assay reliably identified infections during early or peak phases and reduced diagnostic latency, contributing to timely risk assignment.

Routine asymptomatic PCR screening is increasingly questioned due to its limited added value at moderate prevalence and its high cost and effort [6, 20]. Testing residual serum samples proved more logistically feasible and operationally simpler than routine swabbing. This approach can be automated and batched efficiently, offering a practical option where frequent PCR testing is unsustainable. However, a negative antigen result cannot exclude infection, especially after seroconversion, when antigenemia has waned due to antibody neutralization [21]. During the second week of infection, viral loads in sputum decline steadily, leading to N‐protein concentrations below the assay's LoQ [21, 22]. Interpretation of negative findings requires careful attention to timing and complementary diagnostics.

The sensitivity in our cohort (≈60% per sample, ≈30% per patient) was lower than in studies of symptomatic patients sampled early in infection [5, 15, 23], likely due to sampling at varied disease stages, often before systemic antigenemia developed or after it waned. Furthermore, the ELISA platform lacks the sensitivity of advanced single‐molecule assays (> 95%), limiting detection in mild or localized infections [9, 10]. Omicron infections in vaccinated or previously infected patients, with lower viral loads and shorter viremia, also reduced detection rates [15, 24, 25]. As expected, positivity clustered in the early/peak phase (sensitivity ~80%) but declined to 25% in the late phase, confirming that antigenemia reflects systemic replication [9, 10, 23, 26, 27]. Importantly, the estimated comorbidity burden did not differ between true‐positive and false‐negative cases, suggesting that detectability was determined primarily by viral kinetics rather than patient factors.

While PCR remains more sensitive overall, it detects viral RNA and may remain positive beyond the transmissible period. Serum N‐antigen testing may therefore provide complementary information, as detectable antigenemia is more consistent with acute or ongoing infection, particularly in samples with high respiratory viral load or during the early/peak phase of infection. This interpretation is supported by the transient nature of antigenemia and by the marked decline in ELISA sensitivity during the late phase of viral load decrease [5, 10, 14, 27]. However, serum N‐antigen testing may miss infections confined to the upper airways, and neither RT‐PCR viral load nor serum N‐antigen concentration directly proves infectivity, because replication‐competent virus was not assessed by viral culture and no transmission data were available. Thus, serum N‐antigen testing may improve acute‐phase risk classification, but it cannot replace infectivity assays or clinical infection‐control assessment.

Antibody testing must be clearly distinguished from the serum N‐antigen approach evaluated here. Anti‐N antibody positivity, generally induced after SARS‐CoV‐2 infection, can therefore support evidence of previous infection, but it does not establish whether infection is current or whether the patient is infectious. Anti‐N responses may wane over time, but they do not necessarily disappear within days or weeks and may remain detectable for months after infection. Similarly, anti‐spike (S) antibody concentrations are difficult to interpret in vaccinated populations and may reflect vaccination, infection, repeated exposures, or hybrid immunity [28, 29, 30, 31]. Thus, antibody concentrations may be useful for epidemiological classification of prior exposure or immune status, but they are not suitable as standalone markers for acute infection‐control decisions in hospitals.

Adding ELISA to standard PCR reduced unknown‐risk days by ~7% compared to the PCR‐only counterfactual, corresponding to 243 patient‐days with earlier risk classification. In inpatient settings with limited isolation capacity, cohorting options, or staffing resources, reducing the time to risk classification may help decrease avoidable exposure time and support infection‐control workflows.

Persistent antigenemia has been linked to adverse outcomes [32]. In our cohort, deceased patients had higher ECI and were more often antigen‐positive, suggesting that antigenemia could assist in risk stratification. Although not powered for prognosis, this aligns with emerging evidence that systemic antigenemia marks severe disease trajectories.

From a practical perspective, serum‐based testing offers clear advantages. It uses routine blood samples, requires no additional patient contact, and can be batched on ELISA platforms. Unlike RT‐PCR, which requires specialized equipment and trained staff, serum testing can be implemented at scale without overstretching resources. As COVID‐19 becomes endemic and cost pressures increase, widespread PCR screening may not be sustainable, emphasizing the need for cost‐efficient, risk‐adapted, and operationally simple strategies.

Given the shifting epidemiological context, serum antigen testing provides a bridge between comprehensive but costly PCR‐based surveillance and more selective, risk‐based strategies. ELISA detects infections during their most transmissible phase, supporting timely infection control while reducing strain on laboratory capacity. It complements integrated, multi‐layered surveillance systems (targeted PCR testing, serial serum antigen assays, and complementary tools such as wastewater monitoring), expanding coverage and reducing unknown‐risk days at the system level. These benefits support a cost‐effective broad coverage approach where PCR is used for confirmation and outbreak detection.

## 5 Limitations and Future Directions

Our study has several limitations. It was single‐centre, conducted during Omicron predominance near the end of the official pandemic, and based on opportunistic serum availability rather than standardized intervals. Sensitivity estimates therefore reflect pragmatic use rather than theoretical maxima. Further, the lack of exact stability data for the 30–70‐day frozen‐storage interval must be considered as a pre‐analytical limitation.

In addition, the cohort included only hospitalized patients and therefore represents an inpatient population rather than the general population. Admission diagnosis, ward allocation, disease severity, blood‐draw frequency, and hospital‐specific processes may have affected serum availability and infection detection. Consequently, the findings support inpatient surveillance and workflow evaluation, but not unbiased monitoring of emerging pathogens in the wider population.

Anti‐N antibody concentrations were not measured. Therefore, the study cannot assess antibody persistence, hybrid immunity, or the value of serological antibody testing for hospital infection prevention. Future research should prioritize improving the analytical sensitivity of validated serum antigen‐detection platforms, for example, through signal amplification or highly sensitive single‐molecule arrays, to detect lower antigen concentrations [9, 10, 14, 33, 34]. Studies should define optimal sampling intervals and assess integration with pooled PCR and environmental monitoring (e.g., wastewater sampling) to improve early detection. Additionally, multi‐centre studies over longer durations would strengthen the evidence. Evaluating performance in immunocompromised (protracted viral shedding or atypical results) [15] will clarify utility in vulnerable groups. Research could involve quantification of antigenemia to ascertain whether there is a distinction in antigen titre between mild and severe cases and whether adjusting the cutoff value would result in the capture of more cases, albeit at the cost of some false positives. As new variants emerge, ongoing validation will be essential. Development of rapid point‐of‐care serum assays could further expand applications, especially for late presenters. This study underscores the need for highly sensitive, standardized antigen platforms and optimized sampling protocols to capture the dynamic phases of SARS‐CoV‐2 infection. Investigating the role of antigen neutralization kinetics and assay design, particularly antigen‐antibody specificity, should enhance diagnostic accuracy and guide surveillance efforts.

## 6 Conclusions

This study provides evidence from a routine inpatient‐care setting that serum nucleocapsid antigen ELISA, despite limited sensitivity, offers high specificity, practical feasibility, and potential workflow benefits for hospital surveillance. As COVID‐19 becomes endemic with unpredictable seasonal surges, broad PCR screening may no longer be sustainable. Residual serum antigen testing offers a cost‐efficient complement to PCR, preferentially identifying patients during early/high‐replication phases of infection while excluding most convalescent, low‐risk cases. Beyond SARS‐CoV‐2, this approach illustrates how existing hospital laboratory workflows may support scalable inpatient surveillance of emerging pathogens.

## Author Contributions

J.W.: supervision, data curation, formal analysis, methodology, visualisation, writing – original draft, writing – review and editing. A.Z.: data curation, formal analysis, methodology, writing – original draft, writing – review and editing. A.K.: conceptualisation, writing – review and editing. M.P.: conceptualisation, writing – review and editing. A.C.: writing – review and editing. R.W.: conceptualisation, writing – review and editing. M.Ö.: supervision, conceptualisation, data curation, formal analysis, investigation, methodology, visualisation, writing – original draft, writing – review and editing.

## Funding

The authors have nothing to report.

## Ethics Statement

The study was approved by the Ethics Committee of the Medical Faculty, Ruhr University Bochum (22‐7527) and conducted in accordance with the Declaration of Helsinki.

## Conflicts of Interest

The authors declare no conflicts of interest.

## Supporting information


**Figure S1:** SARS‐CoV‐2 inpatient screening distribution by test type, Bochum, Germany, January–February 2023 (n = 2,030 patients; 12,558 patient‐days).
**Figure S2:** Temporal distribution of SARS‐CoV‐2 variants, Bochum, Germany, February 2021–February 2023.

## Data Availability

The data that support the findings of this study are available from the corresponding author upon reasonable request.
