# Supplementary material for: Assessment of Residual‐Serum SARS‐CoV‐2‐N‐Antigen Testing for Hospital Surveillance in Germany
Source: J Med Virol. 2026 Jul 7;98(7):e71055. doi: 10.1002/jmv.71055 (PMC13338776; doi:10.1002/jmv.71055)

**Supplementary Material**

**Supplementary Table S1. Receiver operating characteristic (ROC) analysis of serum SARS-CoV-2 N-antigen concentration for RT-PCR positivity and high viral-load categories.**

| **Endpoint** | **Samples, n** | **Positive endpoint, n** | **AUC** | **Sensitivity, %** | **Specificity, %** | **PPV, %** | **NPV, %** |
| --- | --- | --- | --- | --- | --- | --- | --- |
| RT-PCR positivity | 801 | 99 | 0.803 (0.700–0.882) | 60.6 (40.0–76.5) | 99.9 (99.6–100.0) | 98.4 (93.3–100.0) | 94.7 (92.0–97.0) |
| RT-PCR viral load ≥10⁴ GE/mL | 801 | 77 | 0.889 (0.797–0.948) | 77.9 (59.4–89.7) | 99.9 (99.6–100.0) | 98.4 (93.3–100.0) | 97.7 (96.2–99.0) |
| RT-PCR viral load ≥10⁵ GE/mL | 801 | 58 | 0.936 (0.869–0.975) | 87.9 (75.0–95.6) | 98.7 (97.2–99.7) | 83.6 (65.4–95.5) | 99.1 (98.3–99.7) |

Values are estimates with 95% patient-level bootstrap confidence intervals shown in parentheses. The manufacturer cut-off of 2.97 pg/mL was used as the primary operating point. Numeric serum N-antigen concentrations were used where available; antigen-negative results without numeric concentration were assigned below the cut-off for ROC ordering. The suspected RT-PCR-negative/serum N-antigen-positive artifact was retained in the primary analysis and excluded in sensitivity analysis. AUC, area under the ROC curve; PPV, positive predictive value; NPV, negative predictive value.

**Supplementary Figure 1** **SARS-CoV-2 inpatient screening distribution by test type, Bochum, Germany, January–February 2023 (n = 2,030 patients; 12,558 patient-days)**. The Venn diagram **illustrates** the distribution and overlap of SARS-CoV-2 screening performed using serum antigen testing or nasopharyngeal swab RT-PCR, categorized by the total number of patient days and the number of patients included in each screening modality. Ag: Antigen

**Supplementary Figure 2 Temporal distribution of SARS-CoV-2 variants, Bochum, Germany, February 2021–February 2023**. The stacked area chart shows the relative proportion of circulating variants over time, including Wildtype, Alpha, Delta, and multiple Omicron sublineages (BA.1, BA.2, BA.5, BA.4.6, BA.2.75.2, and BQ.1) based on sequencing data from Knappschaft Kliniken Universitätsklinikum Bochum. The figure illustrates the sequential replacement of dominant variants and the emergence of new Omicron subvariants. During the study period from January 6^th^ 2023 until February 08^th^ 2023 four different Omicron sublineages were prevalent in the hospital.


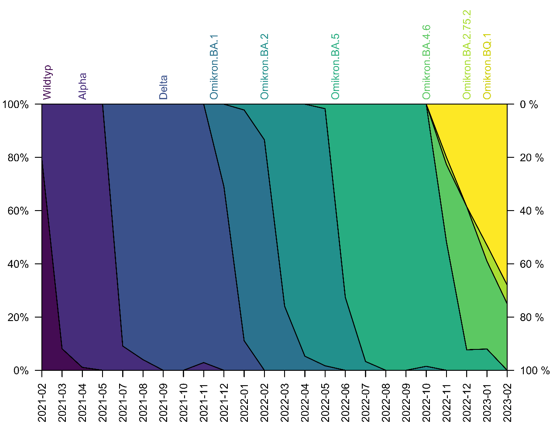

Supplement: Supplementary file 1 — Figure S1: SARS‐CoV‐2 inpatient screening distribution by test type, Bochum, Germany, January–February 2023 (n = 2,030 patients; 12,558 patient‐days). Figure S2: Temporal distribution of SARS‐CoV‐2 variants, Bochum, Germany, February 2021–February 2023. [file JMV-98-e71055-s001.docx]
